# Acute hYpErcapnic respiratory failure in The ICU: A multicenter prospective observational study - The YETI study

**DOI:** 10.1016/j.aicoj.2025.100016

**Published:** 2026-01-16

**Authors:** Claire Dupuis, Toufik Kamel, Christophe Beyls, Charlène Le Moal, Pierre Garcon, Suzanne Goursaud, Laetitia Bodet-Contentin, Alexis Ferré, Max Guillot, Alexandre Gachet, Thibaut Noel, Aude Garin, Laurent Argaud, Konstantinoss Bachoumas, Romain Persichini, Maud Jonas, Mai-Anh Nay, Cyril Cadoz, Frédérique Schortgen, Solène Guinard, Camille Foucault, Guylaine Labro, Jean-François Llitjos, Anahita Rouze, Tài Pham, Bertrand Hermann, the SRLF trial groups

**Affiliations:** a Médecine Intensive-Réanimation, Centre Hospitalo-Universitaire de Clermont-Ferrand, Clermont-Ferrand, France; b Médecine Intensive-Réanimation, Centre Hospitalier Universitaire d’Orléans, Orléans, France; c Réanimation Médico-Chirurgicale Cardio-Thoracique Vasculaire et Respiratoire, CHU Amiens, Amiens, France; d Réanimation Polyvalente, CH Le Mans, Le Mans, France; e Réanimation Polyvalente, GH Est-Francilien, Marne-la-Vallée, France; f Médecine Intensive-Réanimation, Hôpital Côte de Nacre, Caen, France; g Médecine Intensive-Réanimation, INSERM CIC 1415, CRICS-TriGGERSep Network, CHRU de Tours and methodS in Patient-Centered Outcomes and Health ResEarch (SPHERE), INSERM UMR 1246, Université de Tours, Tours, France; h Médecine Intensive-Réanimation, CH de Versailles, Le Chesnay, France; i Médecine Intensive-Réanimation, Hôpital de Hautepierre, CHU de Strasbourg, Strasbourg, France; j Réanimation Polyvalente, CHI Mont-de-Marsan, Mont-de-Marsan, France; k Réanimation Polyvalente, CH Verdun Saint-Mihiel, GHT Cœur Grand Est, Verdun, France; l Médecine Intensive-Réanimation, CH Victor Jousselin, Dreux, France; m Médecine Intensive-Réanimation, Hôpital Édouard Herriot, Lyon, France; n Réanimation Polyvalente, CHD Vendée, La Roche-sur-Yon, France; o Réanimation et Soins-Intensifs Polyvalents, CH de Saintes, Saintes, France; p Médecine Intensive-Réanimation, CH Saint-Nazaire, Saint-Nazaire, France; q Médecine Intensive-Réanimation, CHU Orléans, Orléans, France; r Réanimation Polyvalente, CH Metz, Metz, France; s Réanimation et Surveillance Continue Adulte, Centre Hospitalier Intercommunal de Créteil, Créteil, France; t Réanimation Polyvalente, CHIC Quimper, Quimper, France; u Réanimation Polyvalente, Centre Hospitalier de Cahors, Cahors, France; v Médecine Intensive-Réanimation, GHR Mulhouse et Sud Alsace, Hôpital Emile Muller, Mulhouse, France; w Médecine Intensive-Réanimation, Hôpital Cochin AP-HP, Paris, France; x Médecine Intensive-Réanimation, CHU Lille, Lille, France; y Médecine Intensive-Réanimation, Hôpital de Bicêtre, DMU CORREVE, FHU SEPSIS, Groupe de Recherche Clinique CARMAS, Université Paris-Saclay, AP-HP – Le Kremlin, Icêtre, France; z Université Paris-Saclay, UVSQ, Univ. Paris-Sud, Inserm U1018, Équipe d’Épidémiologie Respiratoire Intégrative, Centre de Recherche en Épidémiologie et Santé des Populations, Villejuif, France; A Université Paris Cité, Institute of Psychiatry and Neurosciences of Paris (IPNP), INSERM U1266, Stroke Team, 75014 Paris, France; B Médecine Intensive-Réanimation, Hôpital Européen Georges-Pompidou (HEGP), AP-HP, Paris, France

**Keywords:** Acute hypercapnic respiratory failure, Intensive care, Ventilatory support

## Abstract

**Background:**

Acute hypercapnic respiratory failure (AHcRF) is a common cause of intensive care unit (ICU) admission, particularly in patients with chronic respiratory diseases. However, large-scale epidemiological data on AHcRF prevalence, management, and outcomes in the ICU remain limited.

**Methods:**

The YETI (Acute hYpErcapnic respiratory failure in The ICU) study was a prospective, multicenter, observational study conducted across 58 ICUs in France and Belgium between December 2021 and June 2022. Adult patients admitted with AHcRF (PaCO₂ > 45 mmHg and clinical signs of respiratory failure) were enrolled. The primary outcome was the prevalence of AHcRF among all ICU admissions. Secondary outcomes included etiologies, management strategies and factors associated with intubation and mortality.

**Results:**

Among 20,482 ICU admissions, 1,010 patients presented AHcRF, and 856 met inclusion criteria. The prevalence for AHcRF among all critically ill patients was 4.9% [95% CI: 4.6, 5.2], and among ICU patients admitted for acute respiratory failure, it was 12.5% [95 % CI: 11.8, 13.2]. Most patients had underlying obstructive lung diseases (Chronic Obstructive Pulmonary Disease: 56.3%; Obstructive Sleep Apnea Syndrome: 18.5%). Infection (52.2%) and cardiogenic pulmonary edema (15.4%) were the most frequent causes for respiratory failure. Non-invasive ventilation (NIV) was used in 81.3% of patients and 35.9% received invasive mechanical ventilation, mainly for coma or NIV failure. ICU mortality was 12.7%. Multivariable analysis identified lower pH, lower Glasgow Coma Scale, infectious etiology, and higher SOFA (Sequential Organ Failure Assessment) score as independent predictors of intubation. ICU mortality was associated with older age, lower body mass index, active cancer, and higher SOFA scores.

**Conclusions:**

Ten percent of patients admitted to the ICU with respiratory failure present AHcRF, primarily driven by chronic obstructive diseases and infections. Despite widespread NIV use, one-third of patients were treated with invasive ventilation. Identifying risk factors for invasive mechanical ventilation and mortality may support earlier triage and personalized management strategies.

## Background

Respiratory failure occurs when the respiratory system is unable to ensure gas exchange, i.e. blood oxygenation and/or decarboxylation. The latter, leading to hypercapnia (defined as an increase in PaCO₂ above 45 mmHg) is commonly referred as type 2 respiratory failure or hypercapnia respiratory failure (AHcRF). Many acute diseases can cause AHcRF and the pathophysiological mechanisms leading to hypercapnia are multifactorial. They include reduced tidal volume, increased dead space, ventilation-perfusion mismatch, decreased minute ventilation and elevated CO₂ production [1,2].

Since the pioneering work by Brochard et al. in the 1990s, which demonstrated that non-invasive ventilation (NIV) reduces both mortality and the need for intubation in patients with acute-exacerbation of chronic obstructive pulmonary disease (AE-COPD) [3,4], the use of NIV has been extended to other conditions, such as acute cardiogenic pulmonary edema (ACPE) and specific neuromuscular diseases associated with acute respiratory failure (ARF).

Most of the available epidemiological data on AHcRF derive from studies assessing the efficacy of NIV in clinical practice [5,6] (Table S1). The interpretation of those studies is challenging due to their limited numbers and the heterogeneity of their results. While approximately 40% of AE-COPD and 10% of ACPE are treated with NIV, data on the use of this treatment in other etiologies are scarce and inconsistent. The etiologies of the remaining AHcRF cases vary widely across studies [[5], [6], [7]], likely reflecting recruitment bias influenced by the expertise of specific centers, as well as the growing use of NIV outside of intensive care units (ICU). Furthermore, the evolving definitions of obesity hypoventilation syndrome (OHS), obstructive sleep apnea syndrome (OSAS), and the more recently recognized asthma-COPD overlap syndrome (ACOS), further complicate the classification of potential comorbidities linked to AHcRF and hinder accurate reporting of the prevalence of NIV use in AHcRF [[8], [9], [10]]. Regarding outcomes, though NIV has significantly reduced mortality in patients with AE-COPD [11] or ACPE [12], these patients are frequently readmitted to the hospital or the ICU, especially among certain COPD populations [13].

In that context, this prospective, observational, multicenter study aimed at characterizing the epidemiological, clinical, and management features of patients admitted to the ICU with AHcRF. We also aimed to identify predictors of invasive mechanical ventilation (IMV) and ICU mortality in the full study cohort.

## Material and methods

### Study design

The YETI (Acute hYpErcapnic respiratory failure in The ICU) study was a multicenter prospective bi-national (Belgium and France) observational study which was designed and conducted by the ‘Commission d’Epidémiologie et de Recherche Clinique’ of the French Intensive Care Society (‘Société de Réanimation en Langue Française’). This study was approved by the institutional review board Comité de Protection des Personnes Est 1 (IRB 2019-A03246-51) and registered on ClinicalTrials.gov (NCT04304339). Informed consent was obtained from each patient or their proxy.

### Study population

All adult patients (≥18 years old) admitted to one of the 58 participating ICUs over a six-month period extending from December 15, 2021, to June 15, 2022, with AHcRF, with or without mechanical ventilation, were eligible. Inclusion criteria were at least one clinical sign of ARF (polypnea >25 breaths per minute, supraclavicular or intercostal retractions, paradoxical thoraco-abdominal breathing); and at least one PaCO_2_ measurement >45 mmHg either drawn before admission or within the first 12 h after ICU admission. The patients were excluded if they were moribund, under guardianship or trusteeship, pregnant or had already been enrolled in the study. Additionally, patients who declined to participate were not included.

### Endpoints

The primary endpoint was the prevalence of AHcRF on ICU admission, defined as the number of patients admitted for AHcRF over the total number of patients admitted to the ICU during the study period.

Secondary endpoints included the etiologies of AHcRF, and main associated comorbidities. Additionally, we aimed to describe the ICU management (including type and duration of ventilatory support) and main outcomes, including ICU length of stay, intubation and ICU mortality.

### Data collection

Data were collected prospectively. Upon admission, the following variables were recorded: (1) baseline characteristics of patients including age, gender, body mass index (BMI), main comorbidities; (2) etiology of AHcRF, such as infection (bacterial and/or viral), AE-COPD, ACPE, pulmonary embolism and drug intoxication; (3) major comorbidities related to AHcRF characterized as obstructive (e.g., COPD, classified according to the Global Initiative for Chronic Obstructive Lung Disease (GOLD) criteria, with a forced expiratory volume in 1 second / forced vital capacity (FEV₁/FVC) ratio <0.7, and other obstructive comorbidities), restrictive (e.g., OHS neuromuscular disorders and other restrictive comorbidities), other lung diseases and heart failure; (4) last pulmonary function tests (when available) including FEV_1_/FVC ratio and total lung capacity; (5) disease severity assessed using the Simplified acute physiology score (SAPS II), the sequential organ failure assessment score (SOFA) and laboratory features including arterial blood gas results; (6) ventilatory support (type -IMV or NIV-, duration and frequency of mechanical ventilation during ICU stay); (7) main outcomes including ICU length of stay (LOS) and ICU death.

### Intubation strategies

Although specific NIV and intubation protocols were not collected from each participating center, intubation was generally considered in cases of worsening acidosis (pH < 7.25 despite optimal NIV), persistent hypoxemia, altered mental status, inability to protect the airway, hemodynamic instability, or NIV intolerance. In practice, persistent hypercapnic coma despite optimal NIV for 30−60 min often led clinicians to proceed with intubation, particularly in patients with isolated hypercapnic acidosis.

### Statistical analysis

We chose to enroll a convenience sample of at least 500 patients to ensure a robust and representative cohort of critically ill patients from France and Belgium.

Descriptive analyses comprised quantitative variables expressed as mean ± standard deviation or median [interquartile (IQR)] and qualitative variables expressed as count and percentage (as appropriate). Proportions were compared using Chi-square (χ²) or Fisher exact tests and continuous variables were compared using Student’s t test or Wilcoxon rank sum test. No imputation of the missing data was performed. For all tests, a two-sided α of 0.05 was considered significant. After carefully checking for non-collinearity among the covariates and selecting them based on expert input, multivariable logistic regression models were constructed—first to identify factors associated with IMV in the whole cohort, and subsequently to determine factors linked to ICU mortality. In the latter model, an interaction between intubation and the Glasgow Coma Scale (GCS) at admission was incorporated. Once the covariates were selected a priori, no additional variable selection procedures were applied. All analyses were performed using R 4.4.2 (The R Foundation for Statistical Computing, Vienna, Austria).

## Results

**Fig. 1:**
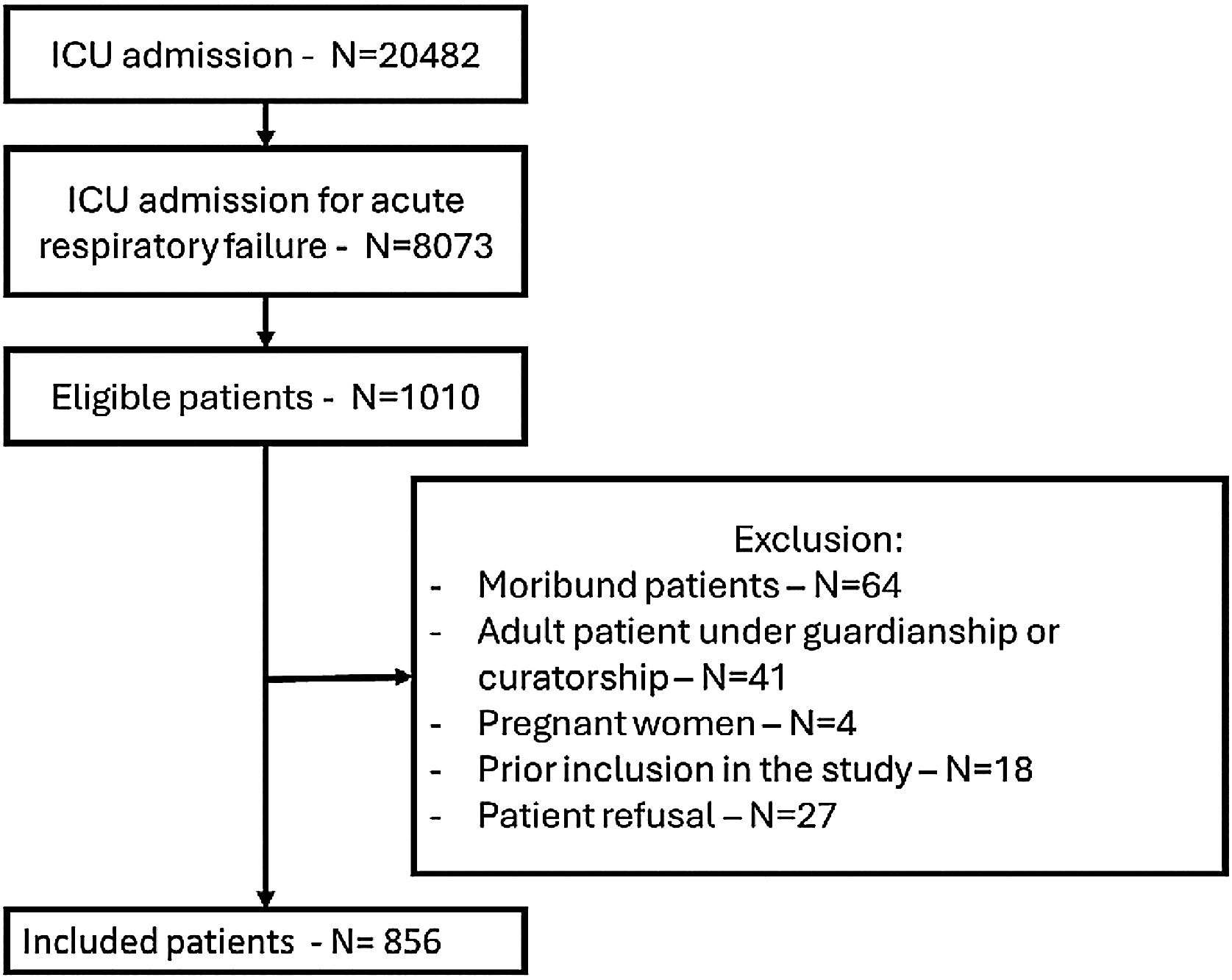
Flow chart. ICU: intensive care unit.

During the study period, 20,482 patients were admitted to the 58 participating centers. Of these, 8,073 presented ARF, and 1,010 AHcRF. A total of 856 patients met all inclusion criteria and no exclusion criteria and were therefore included in the study (Fig. 1).

The total prevalence of ICU admission with AHcRF was 4.9% [95% CI: 4.6, 5.2] among all ICU admissions, and 12.5% [95 % CI: 11.8, 13.2] among ARF ICU patients.

### Overall cohort description

#### Baseline characteristics (Table 1 – Fig. 2)

Most patients were male (62.4%) with a mean age of 68 (±10.7) years, a median [IQR] BMI of 28 kg/m² [22.8–34.6]. A chronic obstructive disease was present in 68.7% of patients, mainly COPD and OSAS (56.3% and 18.5%, respectively). Restrictive lung pathologies were identified in 17.4% of cases, notably OHS (12.6%). A chronic cardiovascular disease was reported in 17.0%, a cancer history in 5.7%, and 56.5% were current or former smokers. Only 28.9% had spirometry values recorded with GOLD classification, and among them, 75.3% patients were reported as GOLD 3 or 4.

**Table 1:** Characteristics on admission in the whole cohort.

Variables	All cohort (N = 856)
Age (mean ± SD) (miss = 14)	66.8 ± 10.7
Gender (Male) (miss = 2)	533 (62.4%)
Body mass index (kg/m²) [Median (IQR)] (miss = 26)	28.0 [22.8; 34.6]
Main Comorbidities in accordance with AHcRF	
Current or former smoking (miss = 7)	480 (56.5%)
Chronic obstructive disease	588 (68.7%)
COPD^a^	482 (56.3%)
>1 AE COPD^b^ (miss = 2)	164/482 (34.0%)
Bronchiectasis	18 (2.10%)
Sleep apnea syndrome	158 (18.5%)
Asthma	64 (7.48%)
Restrictive chronic pathology	149 (17.4%)
Neuromuscular pathology (miss = 1)	19 (2.22%)
Pleural scoliosis surgery (miss = 1)	27 (3.16%)
Obesity syndrome hypoventilation (miss = 1)	108 (12.6%)
Other chronic pulmonary disease (miss = 1)	56 (6.55%)
Cancer (miss = 1)	49 (5.73%)
Bronchial colonization (miss = 1)	7 (0.82%)
Chest trauma (miss = 1)	2 (0.23%)
Chronic cardiovascular disease (miss = 1)	145 (17.0%)
Baseline pulmonary assessment	
FEV1/ FVC^c^ [Median (IQR)] (miss = 609)	51.0 [36.0; 69.0]
Total lung capacity [Median (IQR)] (miss = 695)	104 [80.0; 128]
Usual treatments	
Long-acting Beta-2-mimetics	402 (47.0%)
Long-acting Anticholinergic	303 (35.4%)
Inhaled Steroids	241 (28.2%)
Long term antimicrobial therapy	22 (2.57%)
Long term respiratory support	325 (38.0%)
Aerosols	82 (9.58%)
02 therapy	215 (25.1%)
CPAP and bilevel	173 (20.2%)
Tracheostomy	6 (0.70%)

a: according to the Global Strategy for the Diagnosis, Management, and Prevention of Chronic Obstructive Lung Disease; c: per year; d: Most recent pulmonary function test, regardless of the delay.

COPD: Chronic Obstructive Pulmonary Disease; AE-COPD: Acute Exacerbation of COPD; FEV1/FVC: Forced Expiratory Volume in 1 second / Forced Vital Capacity; CPAP: Continuous Positive Airway Pressure.

**Fig. 2:**
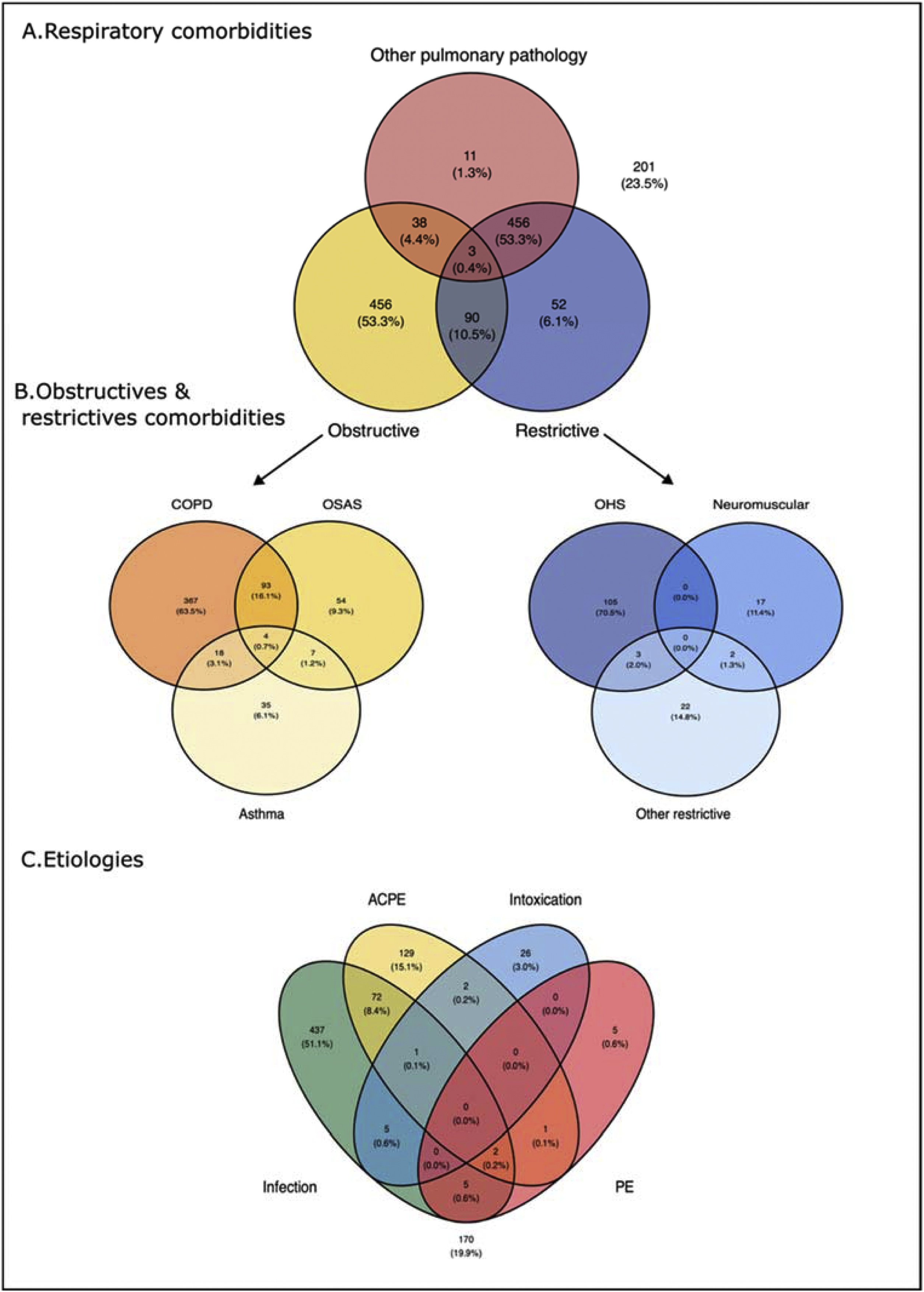
Distribution of Comorbidities and Etiologies in Acute Hypercapnic Respiratory Failure: Venn Diagram Representation. COPD – Chronic Obstructive Pulmonary Disease; OSAS – Obstructive Sleep Apnea Syndrome; OHS – Obesity Hypoventilation Syndrome; ACPE – Acute Cardiogenic Pulmonary Edema; PE – Pulmonary Embolism.

Almost half of the patients (47.0%) received long-acting beta-2 agonists, 35.4% anticholinergics, 28.2% inhaled steroids and 38.0% had long-term respiratory support (oxygen: 25.1%, NIV: 20.2%).

#### Characteristics on admission (Table 2)

The main etiologies of AHcRF were infection (52.2%), ACPE (15.4%) or both (8.8%). The other common etiologies included intoxications (4.0%), pleural effusion (3.2%), pulmonary neoplasia (2.7%), trauma (2.0%), and non-compliance with chronic treatment and/or active smoking (3.0%).

**Table 2:** Main characteristics and interventions at inclusion.

Variables	All cohort (N = 856)
Etiology for AHcRF	
Infection	522 (61.1%)
ACPE	207 (24.2%)
Neither infection/ nor ACPE*	201 (23.5%)
Pulmonary embolism	13 (1.52%)
Drug overdose	34 (3.97%)
Pleural effusion	27 (3.15%)
Ear-Nose-Throat diseases	12 (1.40%)
Pulmonary neoplasia	23 (2.69%)
Trauma	17 (1.99%)
Non-adherence to treatment/Active smoking	26 (3.04%)
Others	41 (4.79%)
Clinical signs of ARF	
Polypnea >25/min	733 (85.6%)
Paradoxical breathing	856 (100%)
Supraclavicular or intercostal pulling	394 (46.0%)
Clinical sign of AHcRF	
Cyanosis (miss = 20)	120 (14.4%)
Sweat (miss = 23)	148 (17.8%)
Flapping tremor (miss = 25)	71 (8.54%)
Severity on inclusion	
pH [Median (IQR)] (miss = 4)	7.27 [7.19; 7.32]
PaCO2 (mmHg) (mean ± SD) (miss = 4)	71.9 ± 22.6
HCO3- (mmol/L) (mean ± SD) (miss = 13)	31.1 ± 7.92
SAPS 2(mean ± SD) (miss = 27)	41.2 ± 16.4
SOFA [Median (IQR)] (miss = 32)	4.00 [2.00; 7.00]
Glasgow Coma scale [Median (IQR)] (miss = 33)	15.0 [12.0; 15.0]
Ventilatory strategies at any time point during ICU stay	
IMV	307 (35.9%)
Time from admission to intubation (hours) [Median (IQR)] (miss = 12)	0.2 [−0.5; 4.3]
Reason for intubation	
Coma	200/307 (65.1%)
High PaCO2	77/307 (25.1%)
Vomiting	1/307 (0.3%)
NIV failure	155/307 (18.1%)
Hypoxemia	123/307 (40.1%)
NIV	696 (81.3%)
Time from admission to 1 st seance of NIV (hours) [Median (IQR)] (miss = 18)	0.2 [0; 4.6]
HFNC	212 (24.8%)
Standard O_2_	575 (67.2%)
Combinations	181 (21.1%)
IMV and NIV	197 (23.0%)
NIV and HFNC	181 (21.1%)
Medical treatments during ICU stay	
Beta-2-mimetics	655 (76.5%)
Anticholinergic	500 (58.4%)
Corticosteroids	411 (48.0 %)
Duration of steroids (days) [Median (IQR)]	5 [3; 6]
Antimicrobial therapy	649 (75.8%)
Diuretics	389 (45.4%)
Transfusion	56 (6.5%)
Others organ supports during ICU stay	
Renal replacement therapy	50 (5.8%)
Vasopressors	244 (28.5%)
vv ECMO	6 (0.7%)

Os; vv ECMO: Venovenous Extracorporeal Membrane Oxygenation.

* Neither INF nor ACPE accounted for the remaining etiologies of AHcRF, which included pulmonary embolism, drug overdose, pleural effusion, and others.

On ICU admission, median GCS was 15 [[12], [13], [14], [15]]. Arterial blood gas analysis showed PaCO₂ of 72 mmHg (±23), pH of 7.27 [7.19–7.32], and HCO₃^−^ of 31 mmol/L (±8). Severity scores were as follows: SAPS II of 41.2 (±16.4), and a SOFA score of 4 [[2], [3], [4], [5], [6], [7]].

#### ICU management and outcomes (Table 2 & Table S2, Fig. 3)

**Fig. 3:**
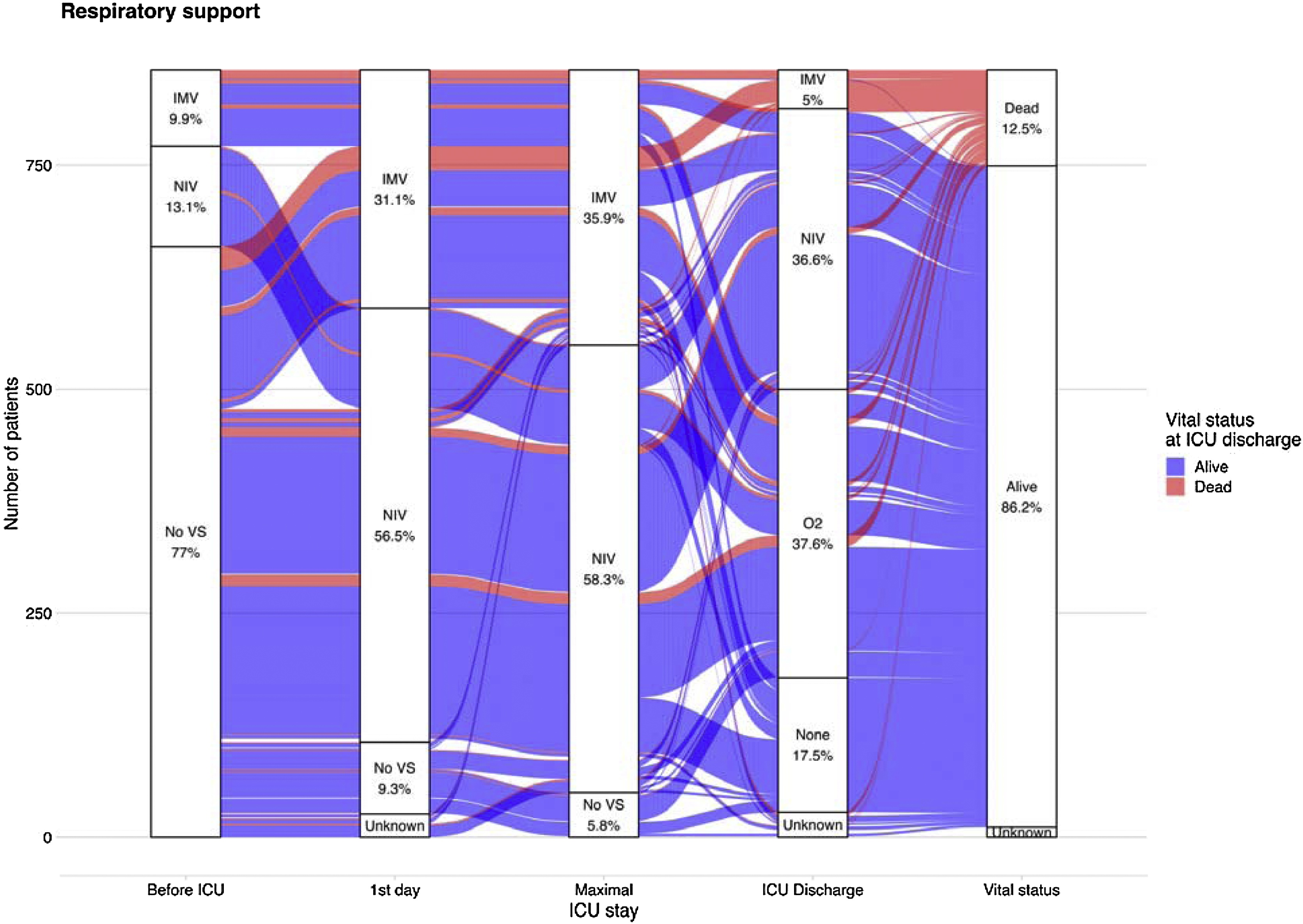
Respiratory Support Trajectories Across ICU Stay in Acute Hypercapnic Respiratory Failure: Alluvial Representation. ICU – Intensive Care Unit; IMV – Invasive Mechanical Ventilation; NIV – Non-Invasive Ventilation; O₂ – Oxygen therapy; VS – Ventilatory support (either IMV or NIV).

During the ICU stay, NIV was used in 81.3% of patients overall, primarily with facial interfaces and for a median duration of 2 [0.7–4.5] days. A total of 307 patients (35.9%) were intubated, including 85 (9.9%) intubated before ICU admission, 181 (21%) intubated within the first 24 h of ICU stay and 41 (4.9%) after 24 h. The main reasons for intubation were coma (65.1%), hypoxemia (40.1%) or high hypercapnia (25.1%). Median duration of IMV was 6 [[2], [3], [4], [5], [6], [7], [8], [9], [10], [11], [12]] days. Among the 197 (28.3%) patients receiving both NIV and IMV, 51.3% had NIV first for a median duration of 5.9 [1.9–25.7] hours. High Flow nasal canula (HFNC) was used in 212 patients (24.8%), either alone (n = 31), or in combination with NIV (n = 181). Extracorporeal CO2 removal (ECCOR) (n = 2) and extracorporeal membrane oxygenation (ECMO) (n = 6) were infrequent. Trajectories of respiratory support across the ICU stay are presented in Fig. 3.

Beta-2 agonists and anticholinergics were administered to 76.5% and 58.4% respectively and steroids (mostly intravenously) were given in 48.0% of the patients for a median duration of 5 [[3], [4], [5], [6]] days. Antimicrobials (75.8%), diuretics (45.5%), and vasopressors (28.5%) were also commonly used.

Overall, 87.3% of patients were discharged alive from ICU. Among them, 57.9% were free of NIV at ICU discharge and 28.3% were weaned from oxygen therapy at ICU discharge. Tracheostomy was performed in 2.3% of the patients. Median ICU length of stay was 6 [[4], [5], [6], [7], [8], [9], [10]] days.

### Comparative analyses

#### Obstructive vs. non-obstructive chronic respiratory failure (Table S3)

Patients with obstructive chronic respiratory failure were older and more frequently on long-term respiratory therapies, as compared with patients with non-obstructive chronic respiratory failure. They received NIV more often (85.4% vs. 72.4%, p < 0.01), while intubation was less frequent (33.8% vs. 40.3%, p < 0.01). Survival was similar in the two groups (84.7% vs. 88.5%, p = 0.16).

#### Comparison according to the etiology for AHcRF (Table S4)

Patients with ACPE were older, had a higher BMI, and more frequently presented with cardiovascular disease, but less often with COPD. At ICU admission, those with both infection and ACPE showed greater severity, with higher SAPS II and SOFA scores, lower pH and GCS values, and higher rates of IMV (all p < 0.01), compared with patients who had only one or no identified etiology. ICU survival did not differ significantly across groups (p = 0.08), although ACPE patients had the highest survival (93.1%). The median ICU length of stay was the longest in the patients with infection (p < 0.01), and successful weaning from non-invasive ventilation at ICU discharge was more frequent in the ACPE group.

#### Intubated vs. Non-Intubated patients (Table S5–S6 & Fig. 4)

In our study, patients who remained non-intubated were older and more frequently had COPD or OHS. Patients who received IMV were more severe with lower pH, higher PaCO₂, lower HCO₃^−^, higher SAPS II and SOFA scores, and lower GCS. Infection was a more common etiology (71.3% vs. 53.0% p < 0.01). ICU stay was longer, tracheostomy more frequent, and survival lower (76.3% vs. 93.5% survival).

**Fig. 4:**
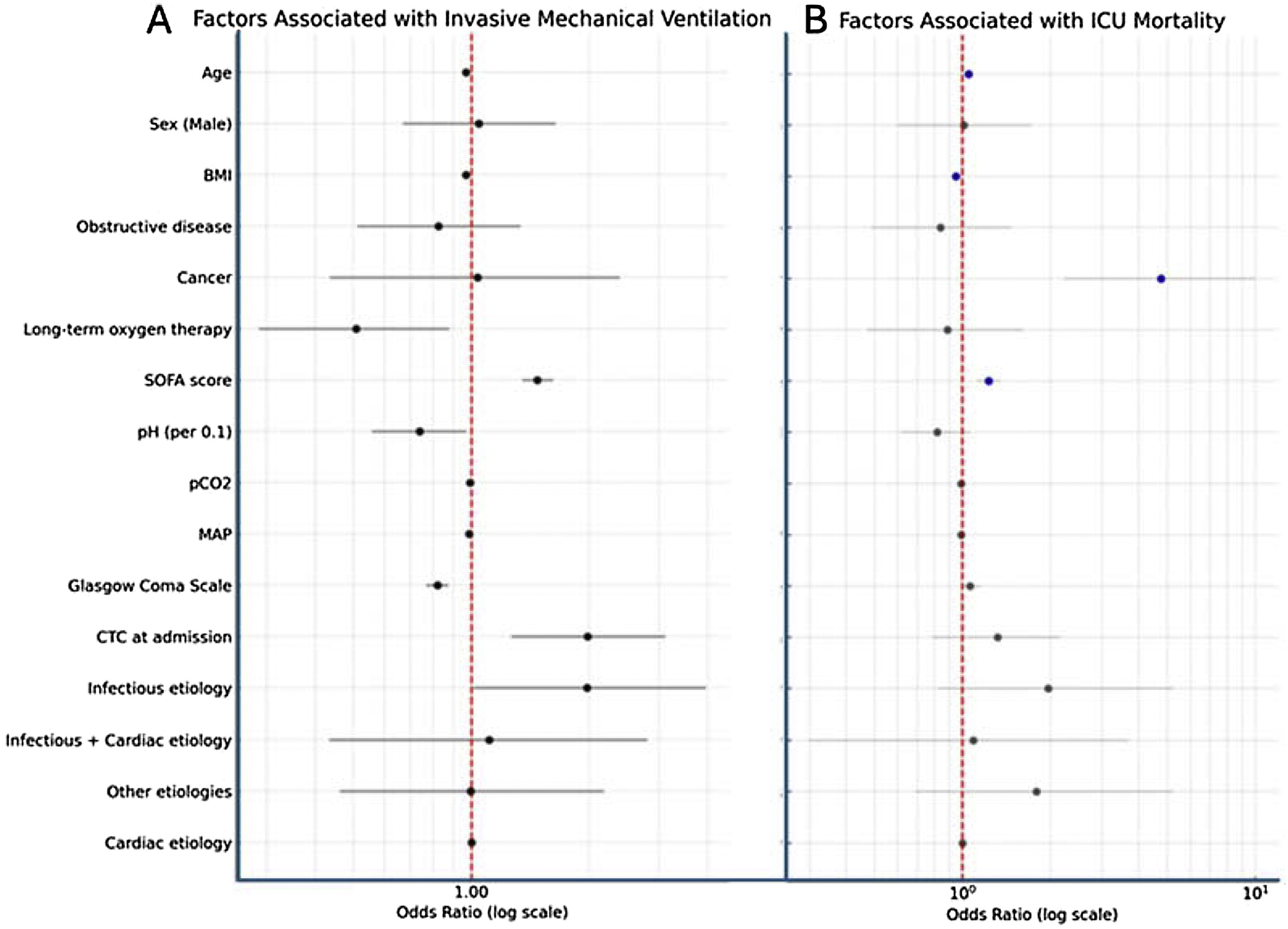
Factors associated with invasive mechanical ventilation (A) and ICU mortality (B), multivariable logistic regression models. BMI– body mass index; CTC – corticosteroids, MAP – mean arterial pressure; SOFA – Sequential organ failure assessment.

The multivariable analysis (Table S6 & Fig. 4) identified several factors significantly associated with IMV. Increasing age (OR = 0.97 per year; 95% CI: [0.95–0.99]; p < 0.01) and higher BMI (OR = 0.97 per kg/m²; 95% CI: [0.94–0.99]; p = 0.01) were associated with a decreased likelihood of intubation. Long-term oxygen therapy was also independently associated with reduced treatment with IMV (OR = 0.51; 95% CI: [0.29–0.88]; p = 0.02). Conversely, higher SOFA scores (OR = 1.47; 95% CI: [1.35–1.62]; p < 0.01), lower pH values (per 0.1 unit decrease, OR = 0.74; 95% CI: [0.56–0.97]; p = 0.03), lower mean arterial pressure (MAP) (OR = 0.99; 95% CI: [0.97–1.00]; p = 0.02), and lower GCS scores (OR = 0.82; 95% CI: [0.77–0.87]; p < 0.01) were significantly associated with increased risk of IMV. Additionally, patients with corticosteroids at admission had significantly higher odds of requiring intubation (OR = 1.98; 95% CI: [1.26–3.12]; p < 0.01), as did those with an infectious etiology (OR = 1.97; 95% CI: [1.01–3.96]; p = 0.05).

#### Survivors vs. non-Survivors (Table 3)

Non-survivors were older, had a lower BMI, and a higher prevalence of cancer compared to survivors. In contrast, OHS was more frequent among survivors. No significant differences were observed regarding the etiologies of AHcRF. Non-survivors also exhibited more pronounced acid-base disturbances at ICU admission, characterized by lower pH and higher bicarbonate levels (HCO₃^−^), and were more frequently treated with IMV. Additionally, ICU length of stay was significantly longer among non-survivors (median 9 [[5], [6], [7], [8], [9], [10], [11], [12], [13], [14], [15]] vs. 6 [[4], [5], [6], [7], [8], [9], [10]] days, p < 0.01).

**Table 3:** Comparison between death and alive patients.

Variables	Non-survivors	Survivors	p-value
Number of patients⚬	N = 107	N = 738	
Age (mean ± SD) (miss = 14)	69.6 ± 10.7	66.3 ± 10.7	0.004
Gender (Male) (miss = 2)	72 (67.3%)	453 (61.5%)	0.292
BMI (kg/m²) [Median (IQR)] (miss = 26)	25.1 [21.2; 31.6]	28.5 [23.1; 34.8]	0.001
Main Comorbidities in accordance with AHcRF			
Current or former smoker (miss = 7)	54 (50.9%)	421 (57.4%)	0.248
Chronic obstructive disease	67 (62.6%)	516 (69.9%)	0.157
COPD^a^	55 (51.4%)	422 (57.2%)	0.307
Restrictive chronic pathology	14 (13.1%)	132 (17.9%)	0.275
Obesity syndrome hypoventilation (miss = 1)	6 (5.61%)	100 (13.6%)	0.031
Cancer (miss = 1)	19 (17.8%)	29 (3.93%)	<0.001
Etiology for AHcRF			
Infection (miss = 2)	75 (70.1%)	442 (60.0%)	0.057
ACPE	17 (15.9%)	187 (25.3%)	0.044
Neither infection/ nor ACPE*	23 (21.5%)	174 (23.6%)	0.718
Usual treatments			
Beta-2-mimetics	41 (38.3%)	355 (48.1%)	0.073
Anticholinergic	34 (31.8%)	264 (35.8%)	0.484
Steroids	25 (23.4%)	212 (28.7%)	0.299
Long term respiratory support	37 (34.6%)	283 (38.3%)	0.519
02 therapy	26 (24.3%)	186 (25.2%)	0.934
CPAP and bilevel	16 (15.0%)	154 (20.9%)	0.195
Tracheostomy	1 (0.93%)	5 (0.68%)	0.557
Severity on inclusion			
pH [Median (IQR)] (miss = 4)	7.23 [7.17; 7.30]	7.27 [7.20; 7.33]	0.015
PaCO2 (mmHg) (mean ± SD)(miss = 4)	72.0 ± 25.4	72.0 ± 22.3	0.985
HCO3- (mmol/L) (mean ± SD)(miss = 13)	29.1 ± 8.05	31.3 ± 7.88	0.010
SAPS 2(mean ± SD) (miss = 27)	55.5 ± 18.7	39.4 ± 15.0	<0.001
SOFA[Median (IQR)] (miss = 32)	6 [4; 10]	4 [2; 6]	<0.001
Glasgow Coma scale [Median (IQR)] (miss = 33)	14.0 [10.0; 15.0]	15.0 [12.5; 15.0]	0.067
Ventilatory strategies during ICU stay			
IMV	72 (67.3%)	232 (31.4%)	<0.001
NIV	72 (67.3%)	616 (83.5%)	<0.001
HFNC	33 (30.8%)	175 (23.7%)	0.139
Medical treatments during ICU stay			
Beta-2-mimetics	71 (66.3%)	574 (77.8%)	.
Anticholinergic	47 (43.9%)	446 (60.4%)	.
Corticosteroids	60 (56.1%)	345 (46.7%)	.
Antimicrobial therapy	97 (90.7%)	546 (74.0%)	.
Transfusion	19 (17.8%)	36 (4.8%)	.
Others organ supports during ICU stay			
Renal replacement therapy	18 (16.8%)	32 (4.3%)	.
Vasopressors	65 (60.7%)	176 (23.8%)	.
ECCOR	1 (0.93%)	1 (0.13%)	
vv ECMO	3 (2.8%)	3 (0.4%)	.
Outcomes			
pH [Median (IQR)] (miss = 4)	7.30 [7.20; 7.40]	7.43 [7.39; 7.46]	<0.001
PaCO2 (mmHg) (mean ± SD)(miss = 4)	59.1 ± 21.0	50.6 ± 12.8	<0.001
HCO3- (mmol/L) (mean ± SD)(miss = 13)	28.7 ± 9.98	32.1 ± 6.18	0.001
Stop NIV at ICU discharge	–	399 (58.0%)	–
Stop O2 at ICU discharge	–	191 (26.8%)	–
Tracheotomy	2 (3.70%)	16 (2.24%)	0.365
ICU LOS [Median (IQR)] (miss = 23)	9 [5; 15]	6 [4; 10]	0.001

⚬ Vital status at ICU discharge was missing in 11 patients. Percentages are thus computed on a total of 845 patients.

IMV: Invasive Mechanical Ventilation; NIV: Non-Invasive Ventilation; HFNC: High-Flow Nasal Cannula; ICU: Intensive Care Unit; LOS: Length of Stay; BMI: Body Mass Index; SD: Standard Deviation; IQR: Interquartile Range; AHcRF: Acute Hypercapnic Respiratory Failure; COPD: Chronic Obstructive Pulmonary Disease; OHS: Obesity Hypoventilation Syndrome; ACPE: Acute Cardiogenic Pulmonary Edema; pH: Potential of Hydrogen (blood acidity/alkalinity); PaCO₂: Partial Pressure of Arterial Carbon Dioxide; HCO₃^−^: Serum Bicarbonate; SAPS II: Simplified Acute Physiology Score II; SOFA: Sequential Organ Failure Assessment; GCS: Glasgow Coma Scale; O₂ therapy: Supplemental Oxygen Therapy; CPAP: Continuous Positive Airway Pressure; ECCOR: Extracorporeal CO₂ Removal; vvECMO: Veno-Venous Extracorporeal Membrane Oxygenation; RRT: Renal Replacement Therapy.

* Neither INF nor ACPE accounted for the remaining etiologies of AHcRF, which included pulmonary embolism, drug overdose, pleural effusion, and others.

The multivariable analysis (Table S7 & Fig. 4) identified older age independently associated with increased mortality (OR 1.05; 95% CI [1.02–1.08]; p < 0.01), while higher BMI was associated with a lower risk mortality (OR 0.95; 95% CI [0.92–0.98]; p < 0.01). The presence of cancer (OR 4.76; 95% CI [2.22–10.00]; p < 0.01) and higher SOFA score at admission (OR 1.23; 95% CI [1.12–1.35]; p < 0.01) were both associated with mortality. These associations remained consistent when accounting for the interaction between intubation and GCS score at admission (Table S8).

## Discussion

In this large, prospective, multicentre cohort study, we provide a comprehensive overview of ICU patients with AHcRF, highlighting current management practices and key prognostic factors for IMV and ICU mortality. The main findings are that: 1) Among patients with acute respiratory failure, AHcRF accounted for only 10% of ICU admissions and COPD and other obstructive lung disorders represented the predominant underlying comorbidities; 2) NIV was the main respiratory support strategy, used in more than 80% of the patients, yet more than one-third of patients were ultimately intubated, primarily due to coma or NIV failure. Multivariable analysis identified older age, lower BMI, active cancer and higher SOFA scores as independent predictors of ICU mortality. Additionally, lower pH, impaired consciousness, and infectious aetiologies were associated with an increased use of IMV.

### Prevalence and patient comorbidities

In this large cohort of more than 20 000 critically ill patients, AHcRF accounted for a relatively small proportion of all ICU admissions (4.2%) and ARF-related ICU admissions (10.6%). To our knowledge, this is one of the first times that such a result has been reported. Indeed, the current literature does not provide precise, large-scale data on the proportion of AHcRF among all ICU admissions or among ICU patients with ARF. Some studies have reported that around 2% of ICU admissions were attributed to COPD-related AHcRF [14] but other studies have emphasized that AHcRF was the cause of ICU admission for around 20% of the patients with acute COPD exacerbation or obesity-related conditions [15,16]. This decrease could be explained in part by the tobacco-control measures. In Europe, sustained tobacco-control measures have contributed to a reduction in COPD prevalence, as documented in Sweden where rates declined in parallel with falling smoking rates [17]. Similarly, the implementation of smoke-free legislation in Spain and or Germany was associated with a significant reduction in COPD-related hospital admissions [18,19]. In addition, advances in pharmacological therapy and the widespread use of non-invasive ventilation during acute exacerbations have decreased the need for intubation and intensive care, allowing more patients to be managed effectively on respiratory wards [19]. In addition, our findings must be interpreted with caution given the particular epidemiological context of our study. Most inclusions occurred during the third and fourth waves of the COVID-19 pandemic, when routine respiratory activity was profoundly disrupted. Widespread mask use, social distancing, and hand hygiene measures markedly reduced the incidence of COPD exacerbations and other respiratory infections [20]. Large national and international datasets consistently reported a sharp decline in hospitalizations for AECOPD during the pandemic [[21], [22], [23]], often accompanied by higher in-hospital mortality

Second, the majority of AHcRF occurred in patients with chronic obstructive respiratory comorbidities, primarily COPD, followed by OHS and OSAS. These results are consistent with earlier reports [[24], [25], [26]]. COPD is present in 60–67 % of the patient but often remain undiagnosed until an acute episode occurs [15,27,28]. Obesity and OSAS are also common, particularly among patients without COPD, with sleep apnea present in up to 81% of cases [15,27,28]. These conditions frequently coexist, with overlap syndromes (such as COPD/OSAS) representing a significant burden and often complicating diagnosis and management [29]. Comorbidities such as obesity and sleep apnea are important risk factors that increase the likelihood of acute decompensation [27], and patients with these overlapping disorders tend to experience more severe nocturnal hypoventilation and have higher rates of ICU admission [30]. Notably, spirometry results were available for only 28.9% of patients in our cohort, suggesting a substantial underdiagnosis. Similarly, Adler et al. previously reported that COPD was unrecognized in two-thirds of AHcRF patients. [15].

Third, our cohort showed a substantial burden cardiovascular disease, present in 17% of patients. Cardiovascular conditions, including hypertension (63–74%) [27,31] and heart disease (49%) [27,31,32], as well as metabolic disorders such as diabetes (24–35%) [27,31,32] and obesity (32–60%) [15,27,28], further worsen outcomes. Other conditions, such as chronic renal failure and immune dysfunction, also contribute to a poorer prognosis in this population. Furthermore, more than half of patients have three or more major comorbidities [15,33,34]. However, we did not collect data on other non-respiratory comorbidities, such as diabetes or chronic renal failure. This is a limitation as non-respiratory comorbidities are highly prevalent in AHcRF patients and significantly impact outcomes.

### Respiratory support

NIV was the cornerstone of AHcRF management, used in approximately 80% of cases. About one quarter of patients were treated with HFNC, while just over one third were ultimately intubated. Extracorporeal circulation, either with ECCO2R or ECMO was unfrequent. These results reflect common clinical practices and are consistent with current guidelines and literature. NIV remains the standard of care for AHcRF, particularly in cases of acute exacerbations of COPD and obesity hypoventilation syndrome [16,35,36]. NIV has been shown to be effective in treating a wide range of disease severities and in various clinical settings, including intensive care units and general wards. It significantly reduces the need for intubation and improves survival in AHcRF patients, including the elderly and those presenting with severe acidosis, compared to standard medical therapy alone [[37], [38], [39]].

HFNC therapy has been proposed as an alternative to NIV, either as initial therapy or in between NIV sessions. However, HFNC has so far demonstrated lower effectiveness than NIV in the initial management of AHcRF, with higher rates of treatment failure and intubation [40,41]. Similarly, ECCO₂R has been explored as a strategy to avoid intubation in patients who fail NIV. Nevertheless, it has not demonstrated any survival benefits and is currently reserved for severe cases requiring highly selective treatment [42,43].

Patients with COPD had a lower intubation rate than other patients (33.8% vs. 40.3%, *p* <  0.01), although the rate remained relatively high compared with other cohorts. For example, in one expert center, the intubation rate among patients with acute-on-chronic respiratory failure managed with NIV was about 15%, with lower rates in cardiogenic pulmonary edema and higher rates in non-COPD etiologies [5]. In a general medical intensive care unit, the NIV failure rate was 19% in COPD patients compared with 47% in non-COPD patients [6].

Regarding factors associated with intubation, our findings indicate that younger age, lower BMI, lower pH, impaired consciousness and infectious etiologies were significantly associated with an increased requirement for intubation. Regarding age, it is likely that older patients were less frequently intubated due to therapeutic limitations. However, we did not collect data to confirm this hypothesis. Patients with obesity were also intubated less often, probably because they respond better to NIV, as did patients with lower pH values at ICU admission. Coma, however, if not rapidly corrected by NIV, constitutes a clear indication for intubation and therefore remains, as expected, an independent predictor of the procedure. These factors have already been reported in the literature. The most consistently reported predictors include high illness severity (as measured by the APACHE II score) [5,6], reduced level of consciousness (low GCS)) [44], severe acidosis and persistent hypoxaemia [5]. Pneumonia has also been reported as a risk factor for intubation [6].

### ICU survival

Overall ICU survival was high (87.3%), particularly in patients with obstructive conditions. Our findings are consistent with Osadnik’s meta-analysis of AECOPD with acute hypercapnic respiratory failure, which showed in-hospital survival of ∼90% with NIV versus ∼82% with usual care (RR for mortality 0.54). [37]. Patients with ACPE had the most favourable outcomes (91.7% survival), which aligns with previous studies reporting consistently low NIV failure in this subgroup [25]. Conversely, intubation was associated with a significantly higher mortality rate (23.7% vs. 6.5% in non-intubated patients, p < 0.01). Recent studies showed high mortality among COPD patients requiring IMV in the ICU with short-term mortality ranging from 20% to 35% [14,45,46], but that may reach 70% in the subgroups treated exclusively with IMV [14]. In a cohort of 670 patients with severe COPD, those ventilated for acute exacerbations had lower ICU mortality (9% vs. 27%; *p* < 0.01) and hospital mortality (17% vs. 32%; *p* = 0.04) compared with other causes of acute respiratory failure [47]. Failure NIV, requiring intubation, is linked to much worse outcomes, with ICU mortality near 60% [48]. International cohorts report ICU mortality from 6% to 28% depending on region, with a decline over time; however, NIV failure consistently predicts poor prognosis [49].

Factors associated with an increased risk of death were older age, lower BMI, cancer and a higher SOFA score. These factors have been highlighted in other studies. The factors most consistently associated with mortality are higher disease severity scores (APACHE II), the need for IMV, severe acidosis, comorbidities (especially cardiac and renal), low functional status and older age [14,37,44]. For example, Akbas et al. reported that cancer and high APACHE II scores, as well as the use of vasopressors, were associated with death [14]. Other studies emphasized the prognostic importance of multimorbidity in AHcRF [15,50]. Similarly, home oxygen use and a higher Charlson comorbidity index are linked to increased mortality [26]. Of note, the association between cancer and ICU mortality should be interpreted with caution, as deaths may be more closely related to an advanced underlying pulmonary condition rather than to cancer itself, a distinction not captured in our study.

### Strengths and limitations

The strengths of this study include its prospective, multicenter design involving 58 ICUs, which enhances generalizability. The sample size (n = 856) and minimal missing data ensured adequate power for subgroup analyses.

However, several limitations must be acknowledged. The collection of spirometry only preceding admission might have led to underdiagnosis of respiratory comorbidities such as OSAS. Reliance on self-reported medical history without confirmatory pulmonary function testing may have also introduced classification bias, especially since the diagnosis of COPD was based on patient-reported information and/or prior medical records, without systematic verification through spirometry, which may have resulted in misclassification or inaccurate prevalence estimates. Long-term outcomes, including post-ICU follow-up data such as hospital readmissions and both short- and long-term post-discharge mortality, were not collected, which limits our understanding of patients’ prognosis beyond the ICU stay — a limitation also noted in previous work by Meservey et al. [26]. Not all therapeutic strategies were investigated, particularly with regard to NIV intensity (investigated in Luo’s HAPPEN trial [51]) and corticosteroid indications. Moreover, awake prone positioning and the use of carbonic anhydrase inhibitors were not assessed. The potential value of clinical scores to guide intubation timing, such as the ROX index, could also have been investigated. Decisions to withdraw or withhold life-sustaining therapies were not collected, despite this being a well-known factor associated with non-intubation orders for patients with chronic respiratory disease. Most inclusions occurred during the COVID-19 pandemic, when preventive measures sharply reduced COPD exacerbations. The low prevalence of AHcRF observed may therefore reflect this exceptional context and may not be generalizable to non-pandemic periods. Although no additional statistical variable selection was performed, this was not necessarily required, as covariates were chosen a priori based on clinical relevance and expert consensus, and further data-driven selection may have added limited value. We acknowledge, however, that some variables may depend on the underlying etiology or disease, such as corticosteroid use. We chose not to perform separate multivariable analyses by underlying condition, as this would have substantially reduced statistical power and increased the risk of model instability given the small subgroup sizes. Moreover, risk factors for intubation were assessed in the entire cohort because only 41 patients were intubated after day 1 post-admission, making subgroup analyses insufficiently robust.

## Conclusions

This large study screening more than 20 000 critically ill patients in France and Belgium found an acute hypercapnic respiratory failure prevalence of 4.2% among all admissions and 12.5% among patients with respiratory failure. NIV remains the cornerstone of its management, being applied in over 80% of patients. Risk stratification should account not only for disease severity but also for factors associated with increased mortality risk—such as age, BMI, GCS, comorbidities, and infection status—to better guide ventilatory management decisions. Future research should focus on long-term outcomes and personalised strategies for this complex patient group.

## CRediT authorship contribution statement

CD, TK, TP, and BH made substantial contributions to the conception of the work. CD, TK, TP, and BH contributed to the study design. All the SRLF trial group contributed to data acquisition. TP performed the analysis. CD, TK, TP, and BH contributed to the interpretation of data. CD and BH drafted the manuscript. All authors revised the manuscript and approved the final version.

## Consent for publication

NA.

## Ethics approval and consent to participate

This study was approved by the institutional review board Comité de Protection des Personnes Est 1 (IRB 2019-A03246-51) and registered on ClinicalTrials.gov (NCT04304339).

## Funding

No funding to declare

## All the past and present members of the CERC (commission épidémiologie et recherche clinique de la SRLF)

Jean-François Llitjos; Laurent Poiroux; Florence Boissier; Sami Hraiech; Lamia Ouanes-Besbes; Gael Piton; Guillaume Decormeille; Gwenaelle Jacq; Saber Barbar; Arnaud Bruyneel; Vanessa Zinzoni; Guillaume Fossat; Simon Bourcier; Nahila Himer; Olivier Lesieur; Gendreau Ségolène; Anaëlle Caillet; Nicolas Fage; Mickael Thy; Antoine Gaillet

We thank Arnaud W. Thille, Nicolas Terzi and Jean-Pierre Frat for their review of the project and/or manuscript.

## Availability of data and material

The data are available from the corresponding author upon reasonable request.

## Declaration of competing interest

No competing of interest to declare
