# Corrigendum to “Acute hYpErcapnic respiratory failure in The ICU: A multicenter prospective observational study - The YETI study” [Ann Intensive Care (2026) 100016]

**DOI:** 10.1016/j.aicoj.2026.100109

**Published:** 2026-07-09

**Authors:** Dupuis Claire, Kamel Toufik, Beyls Christophe, Le Moal Charlène, Garcon Pierre, Goursaud Suzanne, Bodet-Contentin Laetitia, Ferré Alexis, Guillot Max, Gachet Alexandre, Noel Thibaut, Garin Aude, Argaud Laurent, Bachoumas Konstantinoss, Persichini Romain, Jonas Maud, Nay Mai-Anh, Cadoz Cyril, Schortgen Frédérique, Guinard Solène, Foucault Camille, Labro Guylaine, Jean-François Llitjos, Rouze Anahita, Pham Tài, Hermann Bertrand, the SRLF trial groups

**Affiliations:** a Médecine Intensive-Réanimation, Centre Hospitalo-Universitaire de Clermont-Ferrand, Clermont-Ferrand, France; b Médecine Intensive-Réanimation, Centre Hospitalier Universitaire d’Orléans, Orléans, France; c Réanimation Médico-Chirurgicale Cardio-Thoracique Vasculaire et Respiratoire, CHU Amiens, Amiens, France; d Réanimation Polyvalente, CH Le Mans, Le Mans, France; e Réanimation Polyvalente, GH Est-Francilien, Marne-la-Vallée, France; f Médecine Intensive-Réanimation, Hôpital Côte de Nacre, Caen, France; g Médecine Intensive-Réanimation, INSERM CIC 1415, CRICS-TriGGERSep Network, CHRU de Tours and methods in Patient-Centered Outcomes and Health ResEarch (SPHERE), INSERM UMR 1246, Université de Tours, Tours, France; h Médecine Intensive-Réanimation, CH de Versailles, Le Chesnay, France; i Médecine Intensive-Réanimation, Hôpital de Hautepierre, CHU de Strasbourg, Strasbourg, France; j Réanimation Polyvalente, CHI Mont-de-Marsan, Mont-de-Marsan, France; k Réanimation Polyvalente, CH Verdun Saint-Mihiel, GHT Cœur Grand Est, Verdun, France; l Médecine Intensive-Réanimation, CH Victor Jousselin, Dreux, France; m Médecine Intensive-Réanimation, Hôpital Édouard Herriot, Lyon, France; n Réanimation Polyvalente, CHD Vendée, La Roche-sur-Yon, France; o Réanimation et Soins-Intensifs Polyvalents, CH de Saintes, Saintes, France; p Médecine Intensive-Réanimation, CH Saint-Nazaire, Saint-Nazaire, France; q Médecine Intensive-Réanimation, CHU Orléans, Orléans, France; r Réanimation Polyvalente, CH Metz, Metz, France; s Réanimation et Surveillance Continue Adulte, Centre Hospitalier Intercommunal de Créteil, Créteil, France; t Réanimation Polyvalente, CHIC Quimper, Quimper, France; u Réanimation Polyvalente, Centre Hospitalier de Cahors, Cahors, France; v Médecine Intensive-Réanimation, GHR Mulhouse et Sud Alsace, Hôpital Emile Muller, Mulhouse, France; w Médecine Intensive-Réanimation, Hôpital Cochin AP-HP, Paris, France; x Médecine Intensive-Réanimation, CHU Lille, Lille, France; y Médecine Intensive-Réanimation, Hôpital de Bicêtre, DMU CORREVE, FHU SEPSIS, Groupe de Recherche Clinique CARMAS, Université Paris-Saclay, AP-HP – Le Kremlin, Icêtre, France; z Université Paris-Saclay, UVSQ, Univ. Paris-Sud, Inserm U1018, Équipe d’Épidémiologie Respiratoire Intégrative, Centre de Recherche en Épidémiologie et Santé des Populations, Villejuif, France; A Université Paris Cité, Institute of Psychiatry and Neurosciences of Paris (IPNP), INSERM U1266, Stroke Team, 75014 Paris, France; B Médecine Intensive-Réanimation, Hôpital Européen Georges-Pompidou (HEGP), AP-HP, Paris, France

The authors regret that the following sentence “The YETI (Acute hYpErcapnic respiratory failure in The ICU) study was a multicenter prospective bi-national (Belgium and France) observational study […]” was incorrect and should be read as follows instead: “The YETI (Acute hYpErcapnic respiratory failure in The ICU) study was a multicenter prospective multinational (Belgium, France and Morocco) observational study […]”.

The authors also regret that the below contributor was omitted from the Acknowledgements paragraph:

**Rabat** – Khalid Abidi – Medical Intensive Care Unit, Ibn Sina University Hospital Center, Faculty of Medicine and Pharmacy of Rabat, Mohammed V University, Rabat, Morocco; **abidikhalid6@gmail.com**

