# Supplementary material for: Acute hYpErcapnic respiratory failure in The ICU: A multicenter prospective observational study - The YETI study
Source: Ann Intensive Care. 2026 Jan 16;16:100016. doi: 10.1016/j.aicoj.2025.100016 (PMC12934414; doi:10.1016/j.aicoj.2025.100016)
Supplement: Supplementary file 1 [file mmc1.docx]

Table S 1: Main studies dealing with acute hypercapnic respiratory failure admitted in intensive care unit

| Study (Year) | Category | Objective | Population | Key Findings |
| --- | --- | --- | --- | --- |
| Contou (2013) | Prospective | Evaluate NIV outcomes in AHRF and identify predictors of failure | 242 ICU patients with AHRF (PaCO₂ >45 mmHg) | NIV failure varied by etiology (4% CPE to 38% pneumonia). Predictors: pH <7.30, PaO₂/FiO₂ <200 |
| Masa (2016) | Retro | Compare NIV outcomes in AHRF with vs without severe acidosis | 969 AHRF patients (240 ACPE, 540 COPD, 189 OHS) | No difference in NIV failure by acidosis severity; severe acidosis linked to slower pH correction |
| Adler (2017) | Prospective | Characterize undiagnosed comorbidities in AHRF patients | 78 ICU patients with AHRF (COPD/OHS) | 67% had undiagnosed COPD; comorbidities → 46% readmission/death at 3.5 months |
| Akbaş (2023) | Retro | Identify mortality predictors in COPD with AHRF | 100 COPD patients admitted to ICU with AHRF | 90-day mortality 39%; predictors: APACHE II, PaO₂/FiO₂, vasopressors |
| Ghazala (2021) | Retro | Assess NIV success/failure in AECOPD | 370 ICU patients with AECOPD | NIV success 87.3%; failure linked to APACHE III, low BMI |
| Chung (2023) | Case-control | Identify risk factors for HRF | 42 HRF cases vs 105 controls | COPD (PAF 42%), HF, low SNIP, opioids linked to HRF; OSA not significant |
| Chung (2023) | Cross-sectional | Describe comorbidities and mortality in HRF | 873 HRF patients | 83% had comorbidities; mortality 12.8% (↑ with infection, neuromuscular disease) |
| Meservey (2019) | Retro | Analyze readmission/mortality post-HRF | 202 adults hospitalized with HRF | 30-day readmission 23%; post-discharge mortality 36% (↑ with home O₂) |
| Spece (2018) | Retro | Assess comorbidity impact on COPD care | 2,391 veterans hospitalized for COPD exacerbations | Comorbidities reduced guideline-concordant care |
| Kim (2021) | Retro | Evaluate comorbidity impact on exacerbations | 12,000+ COPD patients from Korean database | Comorbidity count independently predicted exacerbations |
| Phua (2005) | Prospective | Compare NIV failure in COPD vs non-COPD AHRF | 111 AHRF patients (COPD vs non-COPD) | NIV failure: COPD 19% vs non-COPD 47% |
| Carrillou (2012) | Retro | Compare NIV in OHS vs COPD-related AHRF | 716 AHRF patients (173 OHS, 543 COPD) | OHS had lower mortality (6% vs 18%) |
| Osadnik (2017) | Meta-analysis | Evaluate NIV efficacy in AECOPD by pH | 17 RCTs of NIV in AECOPD | NIV reduced mortality/intubation across pH ranges |
| Luo (2024) | RCT | Compare NIV intensities in AECOPD | Ongoing RCT of AECOPD patients | Ongoing study |
| Bae (2020) | Retro | Assess HFNC in hypercapnic AHRF | 862 AHRF patients (202 hypercapnic) | HFNC not superior in hypercapnia after adjustment |
| Tan (2024) | RCT | Compare HFNC vs NIV in AECOPD | 225 AECOPD with moderate hypercapnia | HFNC inferior (failure: 25.7% vs 14.3%) |
| Alzaabi (2024) | Retro | Validate NIVO score for AECOPD | 190 AECOPD requiring ventilation | NIVO score predicted NIV failure (AUC 0.85) |
| Gadre (2018) | Retro | Describe outcomes of intubated COPD | 670 intubated COPD patients | ICU mortality 25% (9% in pure AECOPD) |
| Galerneau (2023) | Retro | Assess steroids in AECOPD | 1,247 ICU AECOPD patients | Steroids reduced death/IMV (OR 0.70) but not in severe COPD |
| Akbas (2023) | Retro | describe the clinical course, outcomes, and prognostic factors of COPD with AHcRF | 100 COPD patients with AHcRF in ICU | The main etiologies of AHcRF were bronchitis, pneumonia, and heart failure. The IMV rate was 43%. ICU, in-hospital, and 90-day mortality rates were 21%, 29%, and 39%, respectively. APACHE II scores, the PaO2/FiO2 ratio, vasopressor use, and albumin levels are significant short-term mortality predictors in severely ill COPD patients with AHcRF |

ACPE: Acute Cardiogenic Pulmonary Edema; AECOPD: Acute Exacerbation of Chronic Obstructive Pulmonary Disease; AHcRF: Acute Hypercapnic Respiratory Failure; APACHE II / III: Acute Physiology and Chronic Health Evaluation II / III; AUC: Area Under the Curve; BMI: Body Mass Index; COPD: Chronic Obstructive Pulmonary Disease; FiO₂: Fraction of Inspired Oxygen; HF: Heart Failure; HFNC: High-Flow Nasal Cannula; HRF: Hypercapnic Respiratory Failure; ICU: Intensive Care Unit; IMV: Invasive Mechanical Ventilation; NIVO: Non-Invasive Ventilation Outcomes (score); NIV: Non-Invasive Ventilation; O₂: Oxygen; OHS: Obesity Hypoventilation Syndrome; OR: Odds Ratio; OSA: Obstructive Sleep Apnea; PaCO₂: Arterial Partial Pressure of Carbon Dioxide; PaO₂: Arterial Partial Pressure of Oxygen; PAF: Population Attributable Fraction; pH: Potential of Hydrogen (blood acidity); RCT: Randomized Controlled Trial; SNIP: Sniff Nasal Inspiratory Pressure.

Table S 2: Main outcomes in the whole cohort

| Outcomes | All cohort (N=856) |
| --- | --- |
| Duration of IMV (days) [Median (IQR)] (miss=44) | 6 [2-12] |
| Duration of NIV (days) [Median (IQR)] (miss=91) | 2 [0.7-4.5] |
| At ICU discharge |  |
| pH [Median (IQR)] (miss=18) | 7.42 [7.38;7.46] |
| PaCO2 (mmHg) (mean ± SD) (miss=21) | 51.6±14.3 |
| HCO3- (mmol/L) (mean ± SD) (miss=18) | 31.7±6.85 |
| No NIV at ICU discharge | 361/696 (51.9%) |
| No O2 at ICU discharge | 214 (28.3%) |
| Tracheostomy | 18 (2.33%) |
| ICU LOS (days) [Median (IQR)] (miss=23) | 6 [4;11] |
| Alive at ICU discharge (miss=11) | 738 (87.3%) |

ICU: intensive care unit; IMV: invasive mechanical ventilation; NIV: non-invasive ventilation; LOS: length of stay

Table S 3: Comparison of patients according to Chronic obstructive disease status on ICU admission

| Variables | Non obstruction | Obstruction |  |
| --- | --- | --- | --- |
| Number of patients | N=268 | N=588 | p-value |
| Age (mean ± SD) (miss=14) | 65.2±12.8 | 67.5±9.56 | 0.011 |
| Gender (Male) (miss=2) | 155 (58.1%) | 378 (64.4%) | 0.090 |
| Body mass index (kg/m²) [Median (IQR)] (miss=26) | 28.8 [23.1;35.6] | 27.7 [22.6;34.1] | 0.115 |
| Main Comorbidities in accordance with AHcRF |  |  |  |
| Chronic obstructive disease | 0 (0.00%) | 588 (100%) | <0.001 |
| COPD b | 0 (0.00%) | 482 (82.0%) | <0.001 |
| > 1 AE COPDc (miss=2) | 0 (0.00%) | 164/482 (34.0%) | <0.001 |
| Bronchiectasis | 0 (0.00%) | 18 (3.06%) | 0.008 |
| Sleep apnea syndrome | 0 (0.00%) | 158 (26.9%) | <0.001 |
| Asthma | 0 (0.00%) | 64 (10.9%) | <0.001 |
| Restrictive chronic pathology | 56 (20.9%) | 93 (15.8%) | 0.085 |
| Neuromuscular pathology (miss=1) | 11 (4.10%) | 8 (1.36%) | 0.023 |
| Pleural scoliosis surgery (miss=1) | 16 (5.97%) | 11 (1.87%) | 0.003 |
| Obesity syndrome hypoventilation (miss=1) | 32 (11.9%) | 76 (12.9%) | 0.764 |
| Other chronic pulmonary disease (miss=1) | 15 (5.60%) | 41 (6.98%) | 0.541 |
| Cancer (miss=1) | 13 (4.85%) | 36 (6.13%) | 0.555 |
| Bronchial colonization (miss=1) | 0 (0.00%) | 7 (1.19%) | 0.105 |
| Chest trauma (miss=1) | 0 (0.00%) | 2 (0.34%) | 1.000 |
| Chronic cardiovascular disease (miss=1) | 50 (18.7%) | 95 (16.2%) | 0.426 |
| Etiology for AHcRF |  |  |  |
| Infection (miss=2) | 158 (59.2%) | 364 (61.9%) | 0.495 |
| ACPE | 81 (30.2%) | 126 (21.4%) | 0.007 |
| Neither infection/ nor ACPE | 53 (19.9%) | 148 (25.2%) | 0.107 |
| Usual treatment |  |  |  |
| Beta-2-mimetics | 23 (8.58%) | 379 (64.5%) | <0.001 |
| Anticholinergic | 12 (4.48%) | 291 (49.5%) | <0.001 |
| Steroids | 21 (7.84%) | 220 (37.4%) | <0.001 |
| Long term respiratory support | 47 (17.5%) | 278 (47.3%) | <0.001 |
| 02 therapy | 27 (10.1%) | 188 (32.0%) | <0.001 |
| CPAP and bilevel | 15 (5.60%) | 158 (26.9%) | <0.001 |
| Severity on inclusion |  |  |  |
| pH [Median (IQR)] (miss=4) | 7.27 [7.18;7.34] | 7.27 [7.20;7.32] | 0.906 |
| PaCO2 (mmHg) (mean ± SD) (miss=4) | 70.1±26.2 | 72.6±20.8 | 0.172 |
| HCO3- (mmol/L) (mean ± SD) (miss=13) | 30.0±8.29 | 31.5±7.70 | 0.011 |
| SAPS 2(mean ± SD) (miss=27) | 43.8±17.8 | 40.0±15.5 | 0.004 |
| SOFA[Median (IQR)] (miss=32) | 5 [3;8] | 4 [2;6] | 0.007 |
| Ventilatory supports during ICU stay |  |  |  |
| IMV | 108 (40.3%) | 199 (33.8%) | 0.080 |
| NIV | 194 (72.4%) | 502 (85.4%) | <0.001 |
| HFNC | 72 (26.9%) | 140 (23.8%) | 0.381 |
| Outcomes |  |  |  |
| pH [Median (IQR)] (miss=18) | 7.43 [7.39;7.47] | 7.42 [7.38;7.45] | 0.059 |
| PaCO2 (mmHg) (mean ± SD) (miss=21) | 49.0±13.4 | 52.8±14.6 | <0.001 |
| HCO3- (mmol/L) (mean ± SD) (miss=18) | 30.6±7.13 | 32.2±6.68 | 0.003 |
| Stop NIV at ICU discharge | 138 (66.0%) | 290 (55.4%) | 0.011 |
| Stop O2 at ICU discharge | 84 (37.0%) | 130 (24.6%) | 0.001 |
| Tracheostomy | 11 (4.78%) | 7 (1.29%) | 0.007 |
| ICU LOS [Median (IQR)] (miss=23) | 7 [4;13] | 6 [4;10] | 0.029 |
| Alive at ICU discharge (miss=11) | 222 (84.7%) | 516 (88.5%) | 0.157 |

ACPE: Acute Cardiogenic Pulmonary Edema; AE-COPD: Acute Exacerbation of Chronic Obstructive Pulmonary Disease; AHcRF: Acute Hypercapnic Respiratory Failure; COPD: Chronic Obstructive Pulmonary Disease; CPAP: Continuous Positive Airway Pressure; ICU: Intensive Care Unit; IMV: Invasive Mechanical Ventilation; IQR: Interquartile Range; LOS: Length of Stay; NIV: Non-Invasive Ventilation; O₂: Oxygen; SAPS II: Simplified Acute Physiology Score II; SOFA: Sequential Organ Failure Assessment.

Table S 4: Comparisons according to the etiologies of Acute hypercapnic respiratory failure

| Variables | ACPE | Infectious | Inf & ACPE | Others | P-values | | | | | | |
| --- | --- | --- | --- | --- | --- | --- | --- | --- | --- | --- | --- |
| Number of patients | N=132 | N=447 | N=75 | N=202 | All | ACPE  vs  inf | ACPE  vs  inf & card | ACPE  vs  other | inf  vs  inf & ACPE | inf  vs  other | inf & ACPE  vs  other |
| Age (mean ± SD) (miss=14) | 70.2±10.9 | 65.2±10.4 | 71.1±9.38 | 66.2±10.8 | <0.001 | <0.001 | 0.921 | 0.005 | <0.001 | 0.677 | 0.004 |
| Gender (Male) (miss=2) | 72 (54.5%) | 295 (66.0%) | 46 (61.3%) | 120 (60.0%) | 0.091 | 0.130 | 0.616 | 0.616 | 0.616 | 0.502 | 0.950 |
| Body mass index (kg/m²) [Median (IQR)] (miss=26) | 31.8 [26.5;39.2] | 26.3 [22.0;33.0] | 29.8 [25.5;37.9] | 26.2 [22.0;33.1] | <0.001 | <0.001 | 0.407 | <0.001 | 0.001 | 0.895 | 0.001 |
| Main Comorbidities in accordance with AHcRF |  |  |  |  |  |  |  |  |  |  |  |
| Tobacco usea (miss=7) | 58 (44.6%) | 277 (62.7%) | 38 (50.7%) | 107 (53.0%) | 0.001 | 0.002 | 0.587 | 0.254 | 0.131 | 0.075 | 0.837 |
| Chronic obstructive disease | 76 (57.6%) | 314 (70.2%) | 50 (66.7%) | 148 (73.3%) | 0.017 | 0.026 | 0.509 | 0.025 | 0.625 | 0.586 | 0.527 |
| COPD b | 60 (45.5%) | 268 (60.0%) | 34 (45.3%) | 120 (59.4%) | 0.004 | 0.026 | 1.000 | 0.049 | 0.049 | 1.000 | 0.075 |
| Restrictive chronic pathology | 29 (22.0%) | 80 (17.9%) | 20 (26.7%) | 20 (9.90%) | 0.002 | 0.426 | 0.552 | 0.012 | 0.156 | 0.025 | 0.005 |
| Obesity syndrome hypoventilation (miss=1) | 25 (18.9%) | 51 (11.4%) | 17 (22.7%) | 15 (7.43%) | 0.001 | 0.054 | 0.645 | 0.008 | 0.026 | 0.186 | 0.006 |
| Chronic cardiovascular disease (miss=1) | 58 (43.9%) | 39 (8.74%) | 29 (38.7%) | 19 (9.41%) | <0.001 | <0.001 | 0.664 | <0.001 | <0.001 | 0.901 | <0.001 |
| Etiology for AHcRF |  |  |  |  |  |  |  |  |  |  |  |
| Pulmonary embolism | 1 (0.76%) | 5 (1.12%) | 2 (2.67%) | 5 (2.48%) | 0.377 | 1.000 | 0.598 | 0.614 | 0.598 | 0.598 | 1.000 |
| Medical intoxication | 2 (1.52%) | 5 (1.12%) | 1 (1.33%) | 26 (12.9%) | <0.001 | 0.992 | 1.000 | 0.002 | 1.000 | <0.001 | 0.016 |
| Pleural effusion | 4 (3.03%) | 8 (1.79%) | 3 (4.00%) | 12 (5.94%) | 0.041 | 0.726 | 0.766 | 0.679 | 0.602 | 0.058 | 0.766 |
| Otolaryngological diseases | 0 (0.00%) | 7 (1.57%) | 0 (0.00%) | 5 (2.48%) | 0.241 | 0.600 | . | 0.600 | 0.601 | 0.601 | 0.600 |
| Neoplasia | 0 (0.00%) | 7 (1.57%) | 0 (0.00%) | 16 (7.92%) | <0.001 | 0.450 | . | 0.006 | 0.601 | 0.001 | 0.013 |
| Trauma | 0 (0.00%) | 5 (1.12%) | 0 (0.00%) | 12 (5.94%) | <0.001 | 0.742 | . | 0.011 | 1.000 | 0.005 | 0.067 |
| Non-compliance/Active smoking | 2 (1.52%) | 5 (1.12%) | 1 (1.33%) | 18 (8.91%) | <0.001 | 0.992 | 1.000 | 0.032 | 1.000 | <0.001 | 0.102 |
| Others (miss=22) | 4 (3.03%) | 11 (2.46%) | 0 (0.00%) | 26 (12.9%) | <0.001 | 0.756 | 0.448 | 0.008 | 0.455 | <0.001 | 0.007 |
| Usual treatments |  |  |  |  |  |  |  |  |  |  |  |
| Beta-2-mimetics | 46 (34.8%) | 217 (48.5%) | 35 (46.7%) | 104 (51.5%) | 0.020 | 0.022 | 0.254 | 0.022 | 0.860 | 0.676 | 0.676 |
| Anticholinergic | 28 (21.2%) | 160 (35.8%) | 29 (38.7%) | 86 (42.6%) | 0.001 | 0.007 | 0.022 | 0.001 | 0.727 | 0.178 | 0.727 |
| Steroids | 18 (13.6%) | 126 (28.2%) | 32 (42.7%) | 65 (32.2%) | <0.001 | 0.002 | <0.001 | 0.001 | 0.025 | 0.347 | 0.165 |
| Long term antimicrobial support | 0 (0.00%) | 13 (2.91%) | 4 (5.33%) | 5 (2.48%) |  | 0.233 | 0.098 | 0.322 | 0.467 | 1.000 | 0.388 |
| Long term respiratory support | 45 (34.1%) | 159 (35.6%) | 35 (46.7%) | 86 (42.6%) | 0.104 | 0.834 | 0.212 | 0.226 | 0.212 | 0.212 | 0.763 |
| 02 therapy | 34 (25.8%) | 103 (23.0%) | 20 (26.7%) | 58 (28.7%) | 0.469 | 0.962 | 1.000 | 0.962 | 0.962 | 0.882 | 1.000 |
| CPAP and bilevel | 25 (18.9%) | 84 (18.8%) | 23 (30.7%) | 41 (20.3%) | 0.123 | 1.000 | 0.194 | 1.000 | 0.166 | 1.000 | 0.194 |
| Severity on inclusion |  |  |  |  |  |  |  |  |  |  |  |
| pH [Median (IQR)] (miss=4) | 7.24 [7.17;7.31] | 7.27 [7.19;7.33] | 7.24 [7.17;7.31] | 7.27 [7.20;7.32] | 0.024 | 0.054 | 0.987 | 0.105 | 0.105 | 0.744 | 0.177 |
| PaCO2 (mmHg) (mean ± SD) (miss=4) | 74.2±20.3 | 70.6±21.3 | 74.7±31.8 | 72.2±22.7 | 0.252 | 0.365 | 0.999 | 0.858 | 0.462 | 0.833 | 0.847 |
| HCO3- (mmol/L) (mean ± SD) (miss=13) | 30.7±8.04 | 31.2±12.1 | 31.5±10.7 | 31.2±6.98 | 0.939 | 0.948 | 0.947 | 0.960 | 0.997 | 1.000 | 0.998 |
| SAPS 2(mean ± SD) (miss=27) | 40.2±13.7 | 41.5±17.2 | 46.9±16.5 | 39.0±15.5 | 0.004 | 0.831 | 0.025 | 0.927 | 0.047 | 0.273 | 0.002 |
| SOFA [Median (IQR)] (miss=32) | 4 [3;6] | 4 [2;8] | 5 [3;7.75] | 3 [2;6] | 0.003 | 0.232 | 0.039 | 0.196 | 0.196 | 0.012 | 0.007 |
| Glasgow Coma scale [Median (IQR)] (miss=33) | 15 [13.2;15] | 15 [13;15] | 13 [6;15] | 15 [11;15] | 0.001 | 0.624 | <0.001 | 0.500 | <0.001 | 0.758 | 0.003 |
| Ventilatory support during ICU stay |  |  |  |  |  |  |  |  |  |  |  |
| IMV | 30 (22.7%) | 193 (43.2%) | 30 (40.0%) | 54 (26.7%) | <0.001 | <0.001 | 0.027 | 0.584 | 0.698 | <0.001 | 0.070 |
| NIV | 121 (91.7%) | 347 (77.6%) | 68 (90.7%) | 160 (79.2%) | <0.001 | 0.003 | 1.000 | 0.011 | 0.030 | 0.873 | 0.061 |
| HFNC | 27 (20.5%) | 134 (30.0%) | 15 (20.0%) | 36 (17.8%) | 0.003 | 0.042 | 0.126 | 1.000 | 0.970 | 0.205 | 0.009 |
| Medical treatments during ICU stay |  |  |  |  |  |  |  |  |  |  |  |
| Beta-2-mimetics | 0 (0.0%) | 15 (3.4%) | 3 (4.0%) | 2 (1.0%) | 0.091 | 0.056 | 0.088 | 0.518 | 0.721 | 0.158 | 0.289 |
| Anticholinergic | 60 (45.5%) | 279 (62.4%) | 44 (58.7%) | 117 (57.9%) | 0.006 | 0.002 | 0.062 | 0.047 | 0.516 | 0.484 | 0.928 |
| Corticosteroids | 29 (22.0%) | 256 (57.3%) | 32 (42.7%) | 94 (46.5%) | <0.001 | <0.001 | 0.002 | 0.001 | 0.024 | 0.014 | 0.528 |
| Antimicrobial therapy | 61 (46.2%) | 404 (90.4%) | 66 (88.0%) | 118 (58.4%) | <0.001 | <0.001 | <0.001 | 0.004 | 0.509 | <0.001 | <0.001 |
| Transfusion | 0 (0.0%) | 5 (1.1%) | 0 (0.0%) | 1 (0.5%) | 0.493 | 0.326 | 1.000 | 1.000 | 0.596 | 1.000 | 1.000 |
| Others organ support during ICU stay |  |  |  |  |  |  |  |  |  |  |  |
| Renal replacement therapy | 11 (8.3%) | 26 (5.8%) | 6 (8.0%) | 7 (3.5%) | 0.188 | 0.368 | 0.930 | 0.135 | 0.279 | 0.060 | 0.192 |
| Vasopressors | 22 (16.7%) | 154 (34.5%) | 27 (36.0%) | 41 (20.3%) | <0.001 | <0.001 | 0.001 | 0.524 | 0.784 | 0.002 | 0.001 |
| vv ECMO | 0 (0.0%) | 5 (1.1%) | 0 (0.0%) | 0 (0.0%) | 0.241 | 0.326 | 1.000 | 1.000 | 0.596 | 1.000 | 1.000 |
| Outcomes |  |  |  |  |  |  |  |  |  |  |  |
| pH [Median (IQR)] (miss=18) | 7.42 [7.38;7.46] | 7.42 [7.38;7.46] | 7.43 [7.39;7.48] | 7.42 [7.37;7.45] | 0.210 | 0.624 | 0.940 | 0.355 | 0.451 | 0.325 | 0.449 |
| PaCO2 (mmHg) (mean ± SD) (miss=21) | 53.1±14.9 | 51.1±14.3 | 52.2±13.8 | 51.6±14.3 | 0.586 | 0.686 | 0.533 | 0.974 | 0.795 | 0.937 | 0.985 |
| HCO3- (mmol/L) (mean ± SD) (miss=18) | 32.3±7.00 | 31.5±6.95 | 32.3±6.41 | 31.4±6.72 | 0.521 | 0.454 | 0.651 | 1.000 | 0.637 | 0.814 | 0.997 |
| Stop NIV at ICU discharge | 83 (66.9%) | 218 (58.9%) | 31 (46.3%) | 96 (56.1%) | 0.042 | 0.209 | 0.052 | 0.159 | 0.159 | 0.606 | 0.263 |
| Stop O2 at ICU discharge | 37 (30.1%) | 103 (27.0%) | 15 (22.1%) | 59 (32.4%) | 0.341 | 0.694 | 0.613 | 0.760 | 0.694 | 0.613 | 0.613 |
| Tracheotomy | 1 (0.79%) | 15 (3.77%) | 0 (0.00%) | 2 (1.11%) | 0.079 | 0.285 | 1.000 | 1.000 | 0.285 | 0.285 | 1.000 |
| ICU LOS [Median (IQR)] (miss=23) | 6 [4;9] | 7 [5;13] | 7 [4;10] | 5 [3;10] | <0.001 | 0.001 | 0.152 | 0.864 | 0.412 | <0.001 | 0.152 |
| Alive at ICU discharge (miss=11) | 121 (93.1%) | 376 (84.9%) | 66 (89.2%) | 175 (88.4%) | 0.081 | 0.137 | 0.579 | 0.579 | 0.579 | 0.579 | 1.000 |

ACPE: Acute Cardiogenic Pulmonary Edema; Inf & ACPE : infectious and cardiac etiologies; AHcRF: Acute Hypercapnic Respiratory Failure; BMI: Body Mass Index; COPD: Chronic Obstructive Pulmonary Disease; CPAP: Continuous Positive Airway Pressure; ECMO: Extracorporeal Membrane Oxygenation; FiO₂: Fraction of Inspired Oxygen; HFNC: High-Flow Nasal Cannula; ICU: Intensive Care Unit; IMV: Invasive Mechanical Ventilation; IQR: Interquartile Range; LOS: Length of Stay; NIV: Non-Invasive Ventilation; O₂: Oxygen; PaCO₂: Arterial Partial Pressure of Carbon Dioxide; pH: Potential of Hydrogen (acidity level); SAPS II: Simplified Acute Physiology Score II; SOFA: Sequential Organ Failure Assessment; vv ECMO: Venovenous Extracorporeal Membrane Oxygenation.

Table S 5: Comparison between intubated and non-intubated patients during ICU stay.

| Variables | No IMV | IMV | P-value |
| --- | --- | --- | --- |
| Number of patients | N=549 | N=307 |  |
| Age (mean ± SD) (miss=14) | 67.6±11.0 | 65.3±10.1 | 0.002 |
| Gender (Male) (miss=2) | 328 (60.0%) | 205 (66.8%) | 0.058 |
| BMI (kg/m²) [Median (IQR)] (miss=26) | 28.1 [22.6;35.2] | 27.7 [22.9;33.1] | 0.532 |
| Main Comorbidities in accordance with AHcRF |  |  |  |
| Tobacco use^a^ (miss=7) | 299 (55.1%) | 181 (59.2%) | 0.280 |
| Chronic obstructive disease | 389 (70.9%) | 199 (64.8%) | 0.080 |
| COPD^b^ | 319 (58.1%) | 163 (53.1%) | 0.178 |
| Restrictive chronic pathology | 98 (17.9%) | 51 (16.6%) | 0.716 |
| Obesity syndrome hypoventilation (miss=1) | 73 (13.3%) | 35 (11.4%) | 0.482 |
| Cancer (miss=1) | 29 (5.29%) | 20 (6.51%) | 0.559 |
| Etiology for AHcRF |  |  |  |
| Infection (miss=2) | 299 (54.6%) | 223 (72.6%) | <0.001 |
| ACPE | 147 (26.8%) | 60 (19.5%) | 0.022 |
| Neither infection/ nor ACPE | 147 (26.8%) | 54 (17.6%) | 0.003 |
| Usual treatments |  |  |  |
| Beta-2-mimetics | 266 (48.5%) | 136 (44.3%) | 0.273 |
| Anticholinergic | 208 (37.9%) | 95 (30.9%) | 0.050 |
| Steroids | 154 (28.1%) | 87 (28.3%) | 0.992 |
| Long term respiratory support | 229 (41.7%) | 96 (31.3%) | 0.003 |
| 02 therapy | 160 (29.1%) | 55 (17.9%) | <0.001 |
| CPAP and bilevel | 122 (22.2%) | 51 (16.6%) | 0.061 |
| Tracheostomy | 1 (0.18%)* | 5 (1.63%) | 0.025 |
| Severity on inclusion |  |  |  |
| pH [Median (IQR)] (miss=4) | 7.28 [7.22;7.34] | 7.22 [7.15;7.29] | <0.001 |
| PaCO2 (mmHg) (mean ± SD) (miss=4) | 69.6±19.4 | 75.9±27.0 | <0.001 |
| HCO3- (mmol/L) (mean ± SD) (miss=13) | 32.1±8.11 | 29.1±7.15 | <0.001 |
| SAPS 2(mean ± SD) (miss=27) | 35.3±12.4 | 52.0±17.1 | <0.001 |
| SOFA[Median (IQR)] (miss=32) | 3 [2;4] | 7 [5;10] | <0.001 |
| Glasgow Coma scale [Median (IQR)] (miss=33) | 15 [14 ; 15] | 10 [3;15] | <0.001 |
| Ventilatory strategies during ICU stay |  |  |  |
| IMV | 0 (0.00%) | 307 (100%) | <0.001 |
| NIV | 499 (90.9%) | 197 (64.2%) | <0.001 |
| HFNC | 135 (24.6%) | 77 (25.1%) | 0.939 |
| Medical treatments during ICU stay |  |  |  |
| Beta-2-mimetics | 426 (77.6%) | 229 (74.6%) | . |
| Anticholinergic | 330 (60.1%) | 170 (55.4%) | . |
| Corticosteroids | 238 (43.4%) | 173 (56.4%) | . |
| Antimicrobial therapy | 372 (67.8%) | 277 (90.2%) | . |
| Transfusion | 15 (2.7%) | 41 (13.4%) | . |
| Others organ supports during ICU stay |  |  |  |
| Renal replacement therapy | 14 (2.6%) | 36 (11.7%) | . |
| Vasopressors | 37 (6.7%) | 207 (67.4%) | . |
| ECCOR | 0 (0%) | 2 (100%) |  |
| vv ECMO | 0 (0%) | 6 (2.0%) | . |
| Outcomes |  |  |  |
| pH [Median (IQR)] (miss=4) | 7.42 [7.38;7.45] | 7.43 [7.38;7.47] | 0.036 |
| PaCO2 (mmHg) (mean ± SD) (miss=4) | 53.2±14.0 | 48.9±14.5 | <0.001 |
| HCO3- (mmol/L) (mean ± SD) (miss=13) | 32.8±6.86 | 29.8±6.42 | <0.001 |
| Stop NIV at ICU discharge | 278 (55.9%) | 150 (63.8%) | 0.052 |
| Stop O2 at ICU discharge | 135 (26.4%) | 79 (32.4%) | 0.107 |
| Tracheotomy | 4 (0.78%) | 14 (5.38%) | <0.001 |
| ICU LOS [Median (IQR)] (miss=23) | 5 [3;8] | 11 [7;21] | <0.001 |
| Alive at ICU discharge (miss=11) | 506 (93.5%) | 232 (76.3%) | <0.001 |

ACPE: Acute Cardiogenic Pulmonary Edema; Inf & ACPE : infectious and cardiac etiologies; AHcRF: Acute Hypercapnic Respiratory Failure; BMI: Body Mass Index; COPD: Chronic Obstructive Pulmonary Disease; CPAP: Continuous Positive Airway Pressure; ECMO: Extracorporeal Membrane Oxygenation; FiO₂: Fraction of Inspired Oxygen; HFNC: High-Flow Nasal Cannula; ICU: Intensive Care Unit; IMV: Invasive Mechanical Ventilation; IQR: Interquartile Range; LOS: Length of Stay; NIV: Non-Invasive Ventilation; O₂: Oxygen; PaCO₂: Arterial Partial Pressure of Carbon Dioxide; pH: Potential of Hydrogen (acidity level); SAPS II: Simplified Acute Physiology Score II; SOFA: Sequential Organ Failure Assessment; vv ECMO: Venovenous Extracorporeal Membrane Oxygenation.

Table S 6: Factors associated with invasive mechanical ventilation

| Variable | OR | [95% CI] | p-value |
| --- | --- | --- | --- |
| Age | 0.97 | [0.95 ; 0.99] | <0.01 |
| Sex (Male) | 1.04 | [0.67 ; 1.64] | 0.85 |
| BMI | 0.97 | [0.94 ; 0.99] | 0.01 |
| Obstructive disease | 0.82 | [0.51 ; 1.33] | 0.43 |
| Cancer | 1.04 | [0.43 ; 2.39] | 0.93 |
| Long-term oxygen therapy | 0.51 | [0.29 ; 0.88] | 0.02 |
| SOFA score | 1.47 | [1.35 ; 1.62] | <0.01 |
| pH (per 0.1) | 0.74 | [0.56 ; 0.97] | 0.03 |
| pCO_2_ | 0.99 | [0.98 ; 1.01] | 0.24 |
| MAP | 0.99 | [0.97 ; 1.00] | 0.02 |
| Glasgow Coma Scale | 0.82 | [0.77 ; 0.87] | <0.01 |
| Corticosteroids at admission | 1.98 | [1.26 ; 3.12] | <0.01 |
| Infectious etiology | 1.97 | [1.01 ; 3.96] | 0.05 |
| Infectious + Cardiac etiology | 1.11 | [0.43 ; 2.80] | 0.83 |
| Other etiologies | 0.99 | [0.46 ; 2.17] | 0.99 |
| Cardiac etiology | 1.00 | - | - |

OR: Odds Ratio; 95% CI: 95% Confidence Interval; BMI: Body Mass Index; SOFA: Sequential Organ Failure Assessment; MAP: Mean Arterial Pressure.

Table S 7: Factors associated with ICU mortality

| Variable | OR | [95% CI] | p-value |
| --- | --- | --- | --- |
| Age | 1.05 | [1.02 ; 1.08] | <0.01 |
| Sex (Male) | 1.01 | [0.60 ; 1.72] | 0.98 |
| BMI | 0.95 | [0.92 ; 0.98] | <0.01 |
| Obstructive disease | 0.84 | [0.49 ; 1.47] | 0.54 |
| Cancer | 4.76 | [2.22 ; 10.00] | <0.01 |
| Long-term oxygen therapy | 0.89 | [0.47 ; 1.61] | 0.71 |
| SOFA score | 1.23 | [1.12 ; 1.35] | <0.01 |
| Ph | 0.82 | [0.62 ; 1.06] | 0.15 |
| pCO_2_ | 0.99 | [0.98 ; 1.01] | 0.51 |
| MAP | 0.99 | [0.97 ; 1.00] | 0.06 |
| Glasgow Coma Scale | 1.06 | [0.99 ; 1.15] | 0.09 |
| Corticosteroids at admission | 1.32 | [0.79 ; 2.17] | 0.30 |
| Infectious etiology | 1.96 | [0.83 ; 5.26] | 0.14 |
| Infectious + Cardiac etiology | 1.09 | [0.30 ; 3.70] | 0.89 |
| Other etiologies | 1.79 | [0.69 ; 5.26] | 0.25 |
| Cardiac etiology | 1 | - | - |

OR: Odds Ratio; 95% CI: 95% Confidence Interval; BMI: Body Mass Index; SOFA: Sequential Organ Failure Assessment; MAP: Mean Arterial Pressure.

Table S 8: Multivariable Logistic Regression: OR for ICU Mortality taking into account the interaction between intubation and coma Glasgow scale

| Variable | OR | 95% CI | p-value |
| --- | --- | --- | --- |
| Age | 1.05 | [1.03 ; 1.09] | <0.01 |
| Sex (male) | 1.00 | [0.58 ; 1.72] | 1.00 |
| BMI | 0.95 | [0.92 ; 0.98] | 0.01 |
| Obstructive disease | 0.85 | [0.49 ; 1.49] | 0.57 |
| Cancer | 5.26 | [2.44 ; 12.50] | <0.01 |
| Long-term O₂ therapy | 1.14 | [0.59 ; 2.14] | 0.70 |
| SOFA score | 1.18 | [1.08 ; 1.30] | <0.01 |
| pH | 0.83 | [0.63 ; 1.09] | 0.18 |
| pCO₂ | 0.99 | [0.98 ; 1.01] | 0.48 |
| MAP | 0.99 | [0.97 ; 1.00] | 0.08 |
| Corticosteroids at admission | 1.19 | [0.70 ; 2.00] | 0.52 |
| Infectious etiology | 1.92 | [0.81 ; 5.26] | 0.16 |
| Infectious + cardiac etiology | 1.09 | [0.30 ; 3.80] | 0.89 |
| Other etiologies | 1.69 | [0.64 ; 4.76] | 0.30 |
| Cardiac etiology | 1.00 | — | — |
| Early intubation | 0.29 | [0.04 ; 3.03] | 0.25 |
| GCS | 0.95 | [0.83 ; 1.11] | 0.48 |
| Early intubation : GCS interaction | 1.18 | [0.99 ; 1.37] | 0.04 |

OR: Odds Ratio; 95% CI: 95% Confidence Interval; BMI: Body Mass Index; SOFA: Sequential Organ Failure Assessment; MAP: Mean Arterial Pressure.
